# Mothers’ autonomy and childhood stunting: evidence from semi-urban communities in Lao PDR

**DOI:** 10.1186/s12905-018-0567-3

**Published:** 2018-05-22

**Authors:** Yusuke Kamiya, Marika Nomura, Hina Ogino, Kanako Yoshikawa, Latsamy Siengsounthone, Phonepadith Xangsayarath

**Affiliations:** 1 Faculty of Economics, Ryukoku University, 67 Tsukamoto-cho, Fukakusa, Fushimi-ku, Kyoto, 612-8577 Japan; 2 Japan International Cooperation Agency, 1-6th floor, Nibancho Center Building, 5-25 Niban-cho, Chiyoda-ku, Tokyo, 102-8012 Japan; 3 Mie Prefectural College of Nursing, 1-1-1 Yumegaoka, Tsu-shi, Mie 514-0116 Japan; 4 Osaka School of International Public Policy, Osaka University, 1-31 Machikaneyama, Toyonaka-shi, Osaka, 560-0043 Japan; 5 National Institute of Public Health, Ministry of Health, Samsenthai Road, Ban Kaognot, Sisattanack district, Vientiane Capital, Lao People’s Democratic Republic; 6 National Center for Laboratory and Epidemiology, Ministry of Health, Ban Watnak, Sisattanak district, Vientiane Capital, Lao People’s Democratic Republic

**Keywords:** Women’s autonomy, Mother, Childhood stunting, Undernutrition, Lao PDR

## Abstract

**Background:**

Childhood stunting (height-for-age z-scores below − 2), a form of chronic undernutrition, remains a global health burden. Although a growing literature has examined the association between mothers’ autonomy and childhood stunting, these studies have been limited to countries in South Asia or Sub-Saharan Africa where women have relatively lower social status than do men. Little research has analyzed the effect of mothers’ autonomy on childhood stunting in Lao PDR, where women’s social status is relatively high compared to that in other countries.

**Methods:**

We conducted a cross-sectional questionnaire and body scale measurement targeting 100 mothers and their 115 children (<5 years old) from semi-urban communities in Lao PDR, which is the country with the highest prevalence of childhood stunting in the Indochina region. As dimensions of women’s autonomy, we measured self-esteem, self-efficacy, decision-making power, freedom of mobility, and control of money. We then analyzed how each dimension was associated with the likelihood of childhood stunting.

**Results:**

The likelihood of childhood stunting was significantly lower if mothers had higher self-efficacy for health care (OR = 0.15, *p* = 0.007), self-esteem (OR = 0.11, *p* = 0.025), or control of money (OR = 0.11, *p* = 0.041). In contrast, mothers’ decision-making power and freedom of mobility were not significantly associated with childhood stunting.

**Conclusions:**

We clarified which dimensions of women’s autonomy were associated with childhood stunting in Lao PDR. A closer examination of mothers’ autonomy will aid proper understanding of the determinants of childhood stunting.

## Background

Childhood stunting (height-for-age z-scores below − 2) remains a global health burden, with 156 million stunted children in the world in 2015 [1]. Stunting, a form of chronic undernutrition, is not only harmful to physical growth and cognitive development in the early childhood period but also causes detrimental effects throughout the entire life-cycle, including less educational attainment and lower labor productivity [2–4]. Therefore, reduction in childhood stunting is important to both health and economic development.

The Lao People’s Democratic Republic (Lao PDR) has the highest prevalence of childhood stunting (44.2%) in the Indochina region. Despite Lao PDR’s higher gross national income per capita in 2014 (1660 USD) relative to Cambodia (1020 USD) and Myanmar (1270 USD), its child health and nutrition were inferior to those of neighboring countries [5]. This fact implies the existence of deep-rooted causes specific to Lao PDR that hamper the improvement of children’s health and nutrition.

A proper understanding of the determinants of childhood stunting is extremely important for designing effective policies and programs for children’s health and nutrition. The existing literature has examined the socioeconomic, political, and environmental determinants of childhood stunting in developing countries [6]. Many of these studies have applied the UNICEF’s analytical framework, which categorizes the main causes of child nutritional status into three factors: *immediate*, *underlying*, and *basic* [7]. “Mother”, one of the *underlying* causes, is widely considered to be vital in determining child nutritional status [8]. Previous studies have confirmed that mothers’ demographic factors and improved socioeconomic status, such as age, physical status, literacy, education, employment, and income, contributed to reducing the likelihood of childhood stunting [9, 10].

Additionally, previous empirical studies have shown that mothers’ autonomy plays an important role in determining child nutritional status [8, 9, 11–13]. Here, “autonomy” refers to “the ability… to obtain information and use it as the basis for making decisions about one’s private matters and those of one’s intimates” [14] and “the control women have over their own lives—the extent to which they have an equal voice with their husbands in matters affecting themselves and their families” [15].

Several difficulties exist in measuring women’s autonomy for empirical analysis. First, women’s autonomy is multidimensional [15–18], including social, economic, political, and psychological aspects [18]. Therefore, researchers must determine which aspects of women’s autonomy are positively or negatively associated with children’s health and nutrition. Second, results depend on the context of the study site. Most previous studies on women’s autonomy and child nutrition have been conducted in South Asia and Africa, where women generally have lower social status than men due to, among other factors, social structure. The findings of these studies cannot be generalized to societies in which women can afford higher social status. In Lao PDR, because the majority of the population follows a matrilineal system, women are believed to have relatively high status [19]. Several previous studies have examined the determinants of child nutrition in Lao PDR [20–22], but little research has investigated the role of mothers’ autonomy on childhood stunting. Therefore, the purpose of this study was to measure mothers’ autonomy and assess its association with childhood stunting in Lao PDR.

## Methods

### Study setting

Lao PDR is an ethnically diverse country, with 49 distinct ethnicities and 200 ethnic subgroups [23], and it is one of the few countries in the world in which many ethnic groups follow a matrilineal system [19]. We conducted a field study in semi-urban communities in two districts, Xaythany and Parkngum, of Vientiane Capital of Lao PDR, in August and September 2016. The residents of the targeted communities belonged to the Lao-Tai group, an ethnic majority in Lao PDR that traditionally follows a matrilineal system. In this society, women’s social status is relatively high compared to that of women elsewhere in the world [19]. The underlying reason is that the husband usually moves into the wife’s house after marriage and lives with her parents. The wife, especially if she is the youngest daughter, generally inherits or receives the house, land, and property from her parents [19]. Therefore, it can be hypothesized that wives in this society have greater autonomy inside the household compared to those in other societies.

### Data

Data were collected by a cross-sectional survey. We obtained a household list from the National Institute of Public Health (NIOPH), Ministry of Health, Lao PDR. Considering 19.9% of prevalence of childhood stunting in Vientiane area (Lao Social Indicator Survey 2011–2012 (LSIS) [24]), 95% confidence level, and 8% tolerable error, the minimum sample size was calculated to be 92. Therefore, using simple random sampling, we selected 100 mothers from the list who lived with their husband and at least one child aged 0 to 4 years, and we collected data from all of them (100% response rate). Trained local enumerators collected the data on socio-demographic characteristics of mothers (age, educational level, and height) and children (sex, age, height, and weight) and household asset by a questionnaire survey and body measurement. Information of child’s age was obtained according to their birth date on the family book brought by their mother. Data on household asset was collected based on possession of physical assets, such as radios, televisions, refrigerators, fans, bicycles, and motorbike. Then, we created a composite asset index using a principal component analysis to classify households into four asset quartiles (highest, upper middle, lower middle, lowest). To measure the height and weight of the mothers and children, we used the Seca 213 portable height meter and the Carada Scan HBF-214 weight meter. Experienced researchers from NIOPH supervised the data collection.

### Nutritional status of children

Based on the measured heights and weights, we calculated a height-for-age z-score for each child using WHO Anthro Version 3.2.2. Height-for-age is a longer-term nutritional index that reflects the accumulation of past outcomes and represents the linear growth of a child. A child whose height-for-age is more than two standard deviations below the median of the reference population is classified as “stunted”. Height-for-age is closely related to risk of death for children and is recognized as the optimal indicator for chronic health status [25, 26]. Childhood stunting is therefore considered to be the best indicator of children’s well-being and reflects social inequalities [27].

### Mothers’ autonomy

Considering the multidimensionality of women’s autonomy, we measured five dimensions of mothers’ autonomy: (i) self-efficacy and (ii) self-esteem as psychological aspects, (iii) decision-making power as a familial/interpersonal aspect, (iv) freedom of mobility as a sociocultural aspect, and (v) control of money as an economic aspect [16, 18].

#### Self-efficacy

To measure the degree of mothers’ self-efficacy, we asked two questions: “How easy do you find it to access health services when you need to?” and “How easy do you find it to borrow money when you need to?” We obtained answers on the following Likert scale: “1. Impossible”, “2. Very difficult”, “3. Fairly difficult”, “4. Fairly easy”, and “5. Very easy”. We used a score of 1–5 as the degree of mother’s self-efficacy. For instance, we considered the mother had the highest self-efficacy if her answer was “5. Very easy”.

#### Self-esteem

As a proxy measure of mothers’ self-esteem, we asked about tolerance towards domestic violence. Following the Demographic and Health Survey (DHS) questionnaire [28], we asked the question “Sometimes a husband is annoyed or angered by things that his wife does. In your opinion, is a husband justified in hitting or beating his wife in the following situations” for four different cases: (1) If she goes out without telling him, (2) If she argues with him, (3) If she refuses to have sex with him, and (4) If she does not obey elders in the family. We obtained the answers as “Yes = 0” or “No = 1”. Since women with higher self-esteem would not agree with any justification for a husband hitting or beating his wife, we considered an answer of “No = 1” to indicate high self-esteem. We then constructed a composite score of the answer “No = 1” for four cases as a standardized value (mean = 0, SD = 1).

#### Decision-making power

To measure decision-making power within the household, we asked the question “Who usually decides the following things in your family?” for four different matters: (1) Making purchases for daily household needs, (2) Child health care, (3) Health care for yourself, (4) Visiting your family or relatives. We obtained the answers as “Wife only”, “Husband only”, or “Wife and husband jointly” for each matter. Then, we considered an answer of “Wife only” to indicate high degree of a women’s decision-making power. Finally, we constructed a composite score of the answer “Wife only = 1, or 0 otherwise” for four different matters as a standardized value.

#### Freedom of mobility

To measure the freedom of mothers’ mobility, we asked the question “Do you need to take permission from your husband when you do the following things?” for four different cases: (1) Visit your friends, (2) Go shopping, (3) Go to the clinic or hospital, (4) Go outside the village. We obtained the answers as “Yes = 0” or “No = 1”. We considered an answer of “No = 1” to indicate high freedom of mobility. Similar to the previous two dimensions of autonomy, a composite score of the answer “No = 1” for four different cases was created as a standardized value.

#### Control of money

We asked the question “Do you have money which you can use as you wish?” and obtained the answers as “Yes = 1” or “No = 0”. We considered an answer of “Yes = 1” to indicate high control of money.

Regarding self-esteem, decision-making power, and freedom of mobility, composite scores as a standardized value (mean = 0, SD = 1) were used as explanatory variables in the regression analysis. For each dimension, we confirmed the internal consistency using Cronbach’s alpha.

### Statistical analysis

To estimate the effects of mothers’ autonomy on the likelihood of childhood stunting, we conducted multivariate logistic regression analysis with clustered robust standard errors at the household level to consider correlation among children within the same household. As control variables, we used socio-demographic characteristics of the child (sex and age) and mother (age, education, and height), household asset quartiles, and village dummies. Selection of control variables are based on the UNICEF’s analytical framework on the determinants of child nutrition [7] and empirical studies in Lao PDR [20–22]. Stata version 14 was used as statistical software. In both descriptive and regression analyses, we used 10% as the statistical significance level, not the conventional 5%, by considering a relatively small sample size in this study. In the tables, we reported the significance level either at the 1, 5%, or 10% with asterisks so that the strength of the statistical significance can be easily understood.

## Results

### Mothers’ autonomy

Table 1 shows the results from the questionnaire of mothers’ autonomy. Alongside the mean values of the total sample population, we report the means for mothers who did and did not have stunted children. The mean score of self-efficacy for health services was significantly lower (*p* = 0.067) for mothers with stunted children (=3.7) than for those without stunted children. Regarding self-esteem, 47–74% of mothers answered that a husband is generally not justified in hitting or beating for the four different situations. Mothers without stunted children had higher self-esteem (48–79%) than did mothers with stunted children (43–57%). A statistically significant mean difference was found for “(3) Refuse sex with husband” (*p* = 0.030). Regarding decision-making, more mothers with stunted children made independent decisions than did mothers without stunted children, with statistical significance for “(1) daily household expenses” (*p* = 0.042) and “(2) child health care” (*p* = 0.054). Conversely, mothers without stunted children were more likely make joint decisions with their husbands. For freedom of mobility, 30–47% of mothers answered that they needed permission to go out. Finally, regarding control over money, 38% of mothers answered that they had money that they could use as they wished. No statistical differences were observed between the two groups in terms of freedom of mobility or control over resources.

**Table 1:** Mother’s autonomy

Dimension of autonomy	Total *n* = 100	Stunted *n* = 77	Not stunted *n* = 23	Diff.	*p*-value
Self-efficacy					
Health service	3.9	3.7	4.0	−0.3	0.067*
Borrow money	3.2	3.1	3.3	−0.1	0.513
Self-esteem					
(1) Goes out without telling husband	47%	43%	48%	−5%	0.703
(2) Argues with husband	54%	43%	57%	−14%	0.253
(3) Refuse sex with husband	74%	57%	79%	−23%	0.030**
(4) Not to obey elders	51%	39%	55%	−15%	0.198
Composite score of (1)~(4)	0.57	0.46	0.60	−0.14	0.116
Decision-making power					
(1) Daily household expenses	60%	78%	55%	24%	0.042**
(2) Child health care	42%	48%	40%	8%	0.054*
(3) Health care for wife	58%	74%	53%	21%	0.524
(4) Visit family or relatives	37%	48%	34%	14%	0.224
Composite score of (1)~(4)	0.49	0.62	0.45	0.17	0.083*
Freedom of mobility					
(1) Visit friends	40%	39%	40%	−1%	0.924
(2) Go shopping	47%	48%	47%	1%	0.929
(3) Go to clinic or hospital	37%	39%	36%	3%	0.812
(4) Go outside the village	30%	30%	30%	1%	0.959
Composite score of (1)~(4)	0.39	0.39	0.38	0.01	0.938
Control of money					
Have money to use	38%	35%	39%	−4%	0.720

*p < 0.1, **p < 0.05, *p*-value by t-test

### Sample characteristics

Table 2 shows socio-demographic characteristics of children and their mothers in the sample. The proportion of stunting among 115 children was 21%. According to the LSIS, the prevalence of stunting among children under five in the Vientiane area is 19%. We thus confirmed that no significant difference in the prevalence of stunting existed between our study and the LSIS results. No stunted children in the age category of 0–11 months were present in our sample. Therefore, we did not include children of this age group in the regression analyses.

**Table 2:** Socio-demographic characteristics of children and their mothers

	Frequency	Percentage
Child (*n* = 115)		
Nutritional status		
Stunted	24	21%
Not stunted	91	79%
Sex		
Female	51	44%
Male	64	56%
Age (month)		
0–11	23	20%
12–23	17	15%
24–35	26	23%
36–47	21	18%
48–59	28	24%
Mother (*n* = 100)		
Age (year)		
17–25	25	25%
26–30	29	29%
31–35	32	32%
36–46	14	14%
Educational level		
Preschool/Primary	38	38%
Lower secondary	27	27%
Upper secondary	25	25%
College/University	10	10%

Regarding the mothers’ characteristics, ages ranged from 17 to 46. Mothers whose final educational attainment was preschool or primary were the most common (38%), followed by lower secondary (27%) and upper secondary (25%). Only 10% of mothers finished college or university.

### Regression analysis

Table 3 reports the estimated results from the multivariate logistic regressions. Model 1 shows the results of the basic model, which did not include the dimensions of mothers’ autonomy as explanatory variables. This model shows that girls were less likely to be stunted than boys (OR = 0.13, *p* = 0.004). Higher maternal height reduced the odds of childhood stunting (OR = 0.78, *p* = 0.006). Female dummies and maternal height remained significant throughout the models.

**Table 3:** Results of logistics regressions on the childhood stunting

Outcome variable: whether the child is stunted (=1) or not (=0)							
Model	1	2	3	4	5	6	7
Mother’s autonomy							
Self-efficacy: health		0.15					
		(0.007)***					
Self-efficacy: money			1.05				
			(0.909)				
Self-esteem				0.11			
				(0.025)**			
Decision-making					2.66		
					(0.207)		
Freedom of mobility						1.10	
						(0.902)	
Control of money							0.11
							(0.041)**
Child^a^							
Female	0.13	0.06	0.13	0.09	0.11	0.13	0.08
	(0.004)***	(0.002)***	(0.004)***	(0.001)***	(0.002)***	(0.004)***	(0.015)**
Age (month)							
12–23^b^							
24–35	3.69	2.33	3.74	6.43	4.35	3.72	2.75
	(0.270)	(0.512)	(0.262)	(0.203)	(0.217)	(0.275)	(0.403)
36–47	0.18	0.14	0.18	0.18	0.24	0.18	0.02
	(0.112)	(0.084)*	(0.117)	(0.137)	(0.172)	(0.113)	(0.054)*
48–59	0.75	0.49	0.76	0.80	0.86	0.75	0.28
	(0.770)	(0.510)	(0.786)	(0.841)	(0.877)	(0.774)	(0.364)
Mother							
Age (year)							
17–25^b^							
26–30	1.92	3.36	1.91	2.61	1.73	1.93	4.27
	(0.471)	(0.228)	(0.476)	(0.349)	(0.563)	(0.468)	(0.315)
31–35	0.23	0.48	0.23	0.32	0.29	0.23	0.14
	(0.219)	(0.569)	(0.219)	(0.342)	(0.279)	(0.231)	(0.110)
36–46	0.32	0.48	0.32	0.26	0.35	0.33	0.43
	(0.282)	(0.519)	(0.283)	(0.246)	(0.335)	(0.286)	(0.523)
Educational level							
Preschool/Primary^b^							
Lower secondary	0.24	0.55	0.24	0.26	0.32	0.25	0.10
	(0.106)	(0.517)	(0.117)	(0.138)	(0.219)	(0.111)	(0.048)**
Upper secondary	0.71	2.03	0.71	1.03	0.80	0.73	0.78
	(0.681)	(0.468)	(0.680)	(0.972)	(0.780)	(0.722)	(0.806)
College/University	0.26	0.20	0.26	0.33	0.24	0.26	0.56
	(0.210)	(0.218)	(0.211)	(0.326)	(0.229)	(0.209)	(0.571)
Height	0.78	0.75	0.78	0.73	0.79	0.78	0.73
	(0.006)***	(0.003)***	(0.006)***	(0.001)***	(0.009)***	(0.006)***	(<0.001)***
Household’s asset							
Lowest^b^							
Lower middle	0.73	0.26	0.73	0.66	0.68	0.73	0.49
	(0.727)	(0.163)	(0.728)	(0.674)	(0.701)	(0.728)	(0.549)
Upper middle	0.53	0.33	0.52	1.23	0.47	0.52	0.36
	(0.485)	(0.330)	(0.462)	(0.812)	(0.386)	(0.465)	(0.351)
Highest	0.36	0.20	0.36	0.41	0.39	0.36	0.17
	(0.356)	(0.206)	(0.352)	(0.470)	(0.374)	(0.364)	(0.182)
Observations	92	92	92	92	92	92	92

Estimated coefficients are odds ratio (OR). *p*-value in parenthesis

***p < 0.01, **p < 0.05, *p < 0.1

^a^Children under 12 months are not included in the sample

^b^Reference group, Estimated results for the village dummies are omitted from the table

Models 2–7 present how each dimension of mothers’ autonomy was associated with the likelihood of childhood stunting. First, mothers’ higher self-efficacy for accessing health services lowered the odds of childhood stunting (OR = 0.15, *p* = 0.007). However, the effect of mothers’ self-efficacy for borrowing money was not statistically significant. Second, higher self-esteem was related to a lower likelihood of childhood stunting (OR = 0.11, *p* = 0.025). Third, the degree of mothers’ sole decision-making was not significantly associated with childhood stunting. Fourth, mothers’ freedom of mobility was not significantly associated with childhood stunting. Finally, regarding control over money, if mothers had income earned independently, their children were significantly less likely to be stunted (OR = 0.11, *p* = 0.041).

## Discussion

We examined how mothers’ autonomy was associated with the likelihood of childhood stunting by examining original survey data gathered from four semi-urban communities in Vientiane Capital of Lao PDR. Specifically, we categorized mothers’ autonomy into five dimensions: self-efficacy, self-esteem, decision-making, freedom of mobility, and control over money. We then examined how each dimension was associated with the likelihood of childhood stunting by multivariate logistic regression analysis.

First, we confirmed that mothers’ higher self-efficacy for accessing health services was significantly associated with lower likelihood of childhood stunting. This finding suggests that if a mother has greater confidence in accessing health services, her child is less likely to be stunted. Similar results have been found in other countries. For example, mothers’ lower autonomy over medical treatment for their children during periods of illness was associated with negative child growth in an urban slum of Bombay, India [29]. In rural Chad, mothers’ higher influence on child feeding was positively related with children’s height-for-age [30]. However, we did not find any significant effect of mothers’ self-efficacy for borrowing money on childhood stunting in our sample. One possible explanation is that money borrowing was uncommon among mothers in our study site as we confirmed that 38% of the mothers had money to use. Thus, mothers’ confidence over money borrowing was unrelated to children’s health.

Second, we confirmed that mothers’ higher self-esteem, as measured by intolerance toward domestic violence by a husband, was significantly associated with reduced likelihood of childhood stunting. This result is consistent with that of a previous study in PDR using nationally representative data, which confirmed a statistically significant association between mothers’ intolerance toward domestic violence and children’s higher height-for-age [22]. In contrast, mothers’ attitude towards domestic violence had no association with childhood stunting in India [31] and Tanzania [32]. The above results thus suggest a positive association between maternal self-esteem and child linear growth in the matrilineal society of Lao PDR. Among individual components of mother’s self-esteem, a statistically significant difference between mothers with stunted children and those with non-stunted children was confirmed for “(3) Refuse sex with husband” (Table 1). Since this component is directly related to marital sexual relationship, the result implies that mother’s higher autonomy in terms of sexuality behavior with a husband would lead to the improvement of child growth.

Third, the degree of mothers’ sole decision-making was not associated with the likelihood of childhood stunting. This result suggests that child linear growth is not affected by who makes decisions in the household. Empirical studies in other countries have yielded mixed results on this point. For example, a cross-country study that analyzed DHS datasets from 12 developing countries found that mothers’ greater decision-making power was positively related to children’s height-for-age in India and Mali. Conversely, women’s decision-making power was negatively associated with height-for-age in Haiti and Malawi [33]. As decision-making variables, this study examined whether the woman had the final say in health care, making large household purchases, household purchases for daily needs, and visits to family or relatives. Regarding decision-making on healthcare, mothers’ higher autonomy in choosing their own health care was associated with improved height-for-age among children in Tanzania [32]. Similarly, whether mothers had the final say on their own health care was associated with lower likelihood of childhood stunting in rural Nepal [34]. However, no significant association was found between any of the household decision-making variables (cooking, healthcare, purchasing, and going out) and childhood stunting in India [31]. Laotian women in the matrilineal system are generally afforded greater decision-making power than those in other societies [35]. Our study findings showed that mothers’ sole decision-making was not associated with childhood stunting in this society.

Absence of significant effect of mother’s sole decision-making on the childhood stunting implies shared decision-making between wife and husband may be more beneficial with regard to child growth. To scrutinize this point, we constructed a composite score of the answer “Wife and husband jointly = 1, or 0 otherwise” for four different matters with regard to decision-making. Then, we used this composite score as an explanatory variable in the multivariate logistic regression and checked its effect on childhood stunting. The result shows that although direction of the estimated coefficient for shared decision-making was toward a reduction in the childhood stunting, its statistical significance was not confirmed. We need further investigation on what forms of decision-making between wife and husband would be more favorable in terms of child growth.

Fourth, mothers’ freedom of mobility was not a statistically significant determinant of childhood stunting. Laotian women in matrilineal society have less limitation in going out compared to those in South Asia, which likely affected this result. A study from India showed that mothers who did not need permission to go to the market were less likely to have a stunted child [31]. In contrast, no significant relationship between mothers’ mobility and children’s height-for-age was found in Egypt [36].

Fifth, the children in our sample were less likely to be stunted if their mothers had independent income. This result suggests that mothers who can use money as they wish are more likely to spend money on their child’s welfare. Similar results were found in South Asia. In India, whether mothers were allowed to set aside money for personal use was associated with reduced likelihood of childhood stunting [31]. In Nepal, mothers’ control over income was associated with higher height-for-age of their children [37]. Our study suggests that mothers’ financial resources are important for children’s growth regardless of women’s social status in different societies.

Finally, regarding mothers’ basic characteristics, we found that mothers’ height was significantly and positively associated with the likelihood of childhood stunting. This result is consistent with results from other developing countries. For example, children were more likely to be stunted if their mothers were lower height or underweight (Body Mass Index <18.5 kg/m^2^) in Kenya [38], Tanzania [39], and Sri Lanka [40]. Because little previous work has examined the association between mothers’ physical state and childhood stunting in PDR, our study will provide important input for future research.

Recently, an increasing number of studies have been conducted to examine the association between women’s autonomy and children’s health in developing countries. However, very few studies have examined the effects of women’s autonomy on children’s health for societies in which women have relatively higher status than men, such as Laotian matrilineal society. Therefore, our analysis provides new findings in this study area.

Our research had several limitations. First, the direction of causality from mothers’ autonomy to children’s health outcomes was not fully confirmed due to the nature of the cross-sectional data. Further study will be required to investigate this causality. Second, we could not identify the concrete reasons behind the association of mothers’ autonomy and childhood stunting. Future research should shed light on the mechanism behind this relationship. Third, our survey was conducted in restricted areas of Vientiane Capital, and thus the study results cannot be generalized to the entire Lao population. Despite these limitations, this study is the first to analyze the association between mothers’ autonomy and childhood stunting in Lao PDR, and it will thus serve as an important benchmark for further work.

## Conclusions

We clarified which dimensions of women’s autonomy were associated with childhood stunting in Lao PDR. A closer examination of mothers’ autonomy will aid proper understanding of the determinants of childhood stunting.

## Abbreviations

DHS: Demographic and health survey
Lao PDR: Lao People’s Democratic Republic
LSIS: Lao social indicator survey 2011–2012
NIOPH: National Institute of Public Health
OR: Odds ratio
